# Supplementary material for: Phylogenomic analyses of all species of swordtail fishes (genus Xiphophorus) show that hybridization preceded speciation
Source: Nat Commun. 2024 Aug 4;15:6609. doi: 10.1038/s41467-024-50852-6 (PMC11298535; doi:10.1038/s41467-024-50852-6)
Supplement: Supplementary file 1 — Supplementary Information [file 41467_2024_50852_MOESM1_ESM.pdf]

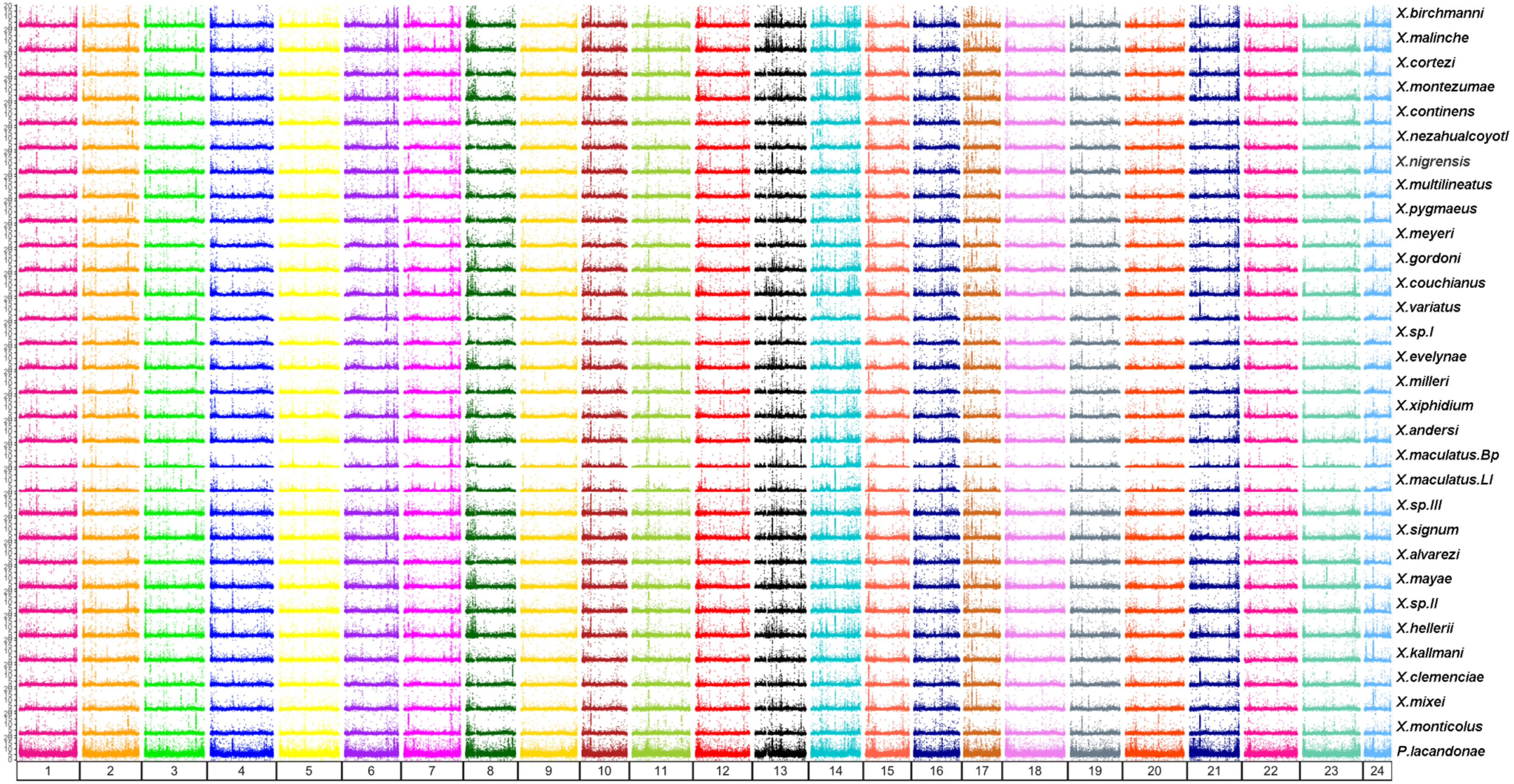

Supplementary figure S1. Plots displaying sequence differences between *X. maculatus* and all other species across chromosomes. Sequence difference was calculated in 10kb sliding windows as the percentage of SNP and indels.

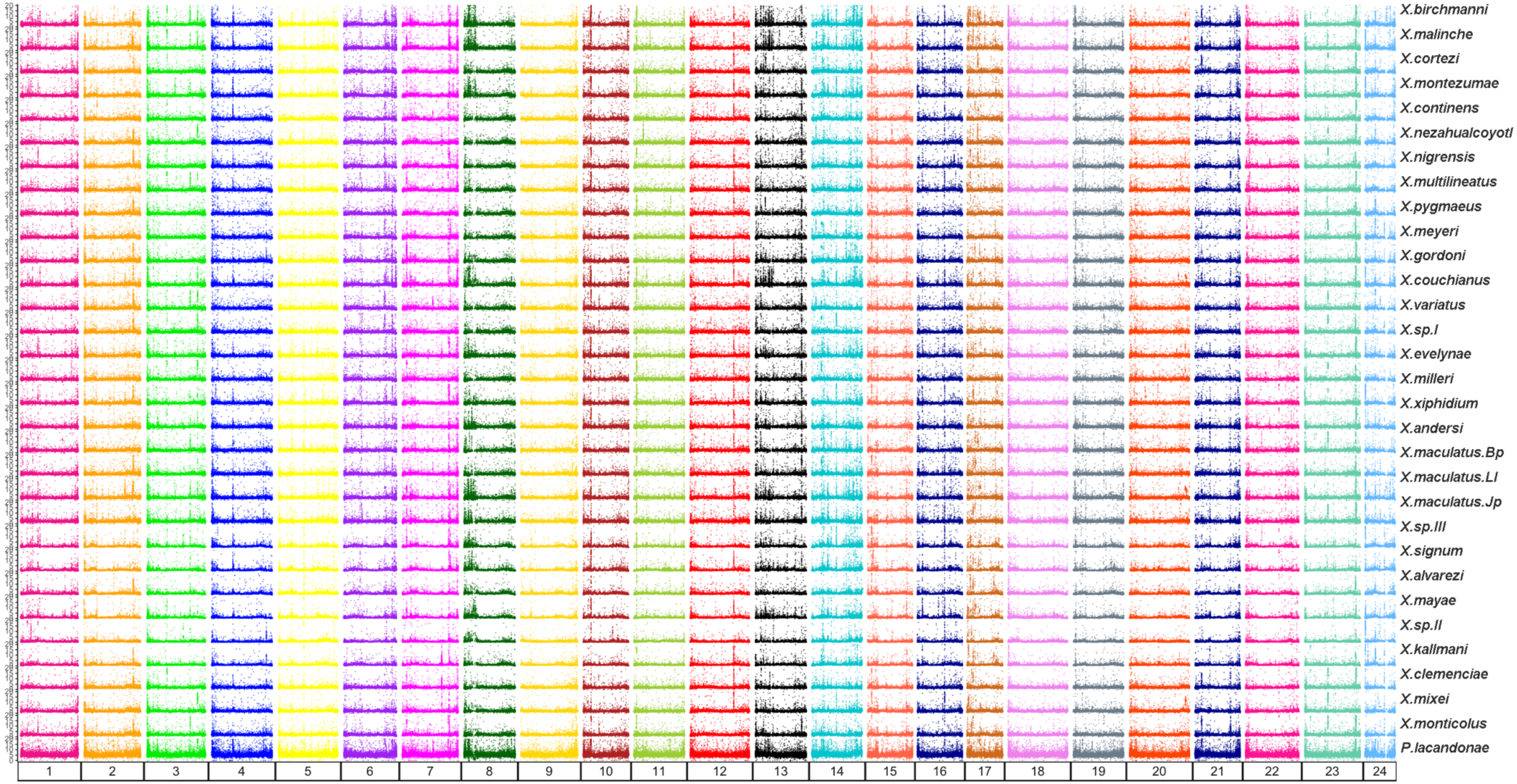

Supplementary figure S2. Plots displaying sequence differences between *X. hellerii* and all other species across chromosomes. Sequence difference was calculated in 10kb sliding windows as the percentage of SNP and indels. Note the high similarity of patterns between species and the analysis in Supplementary Figure S1.

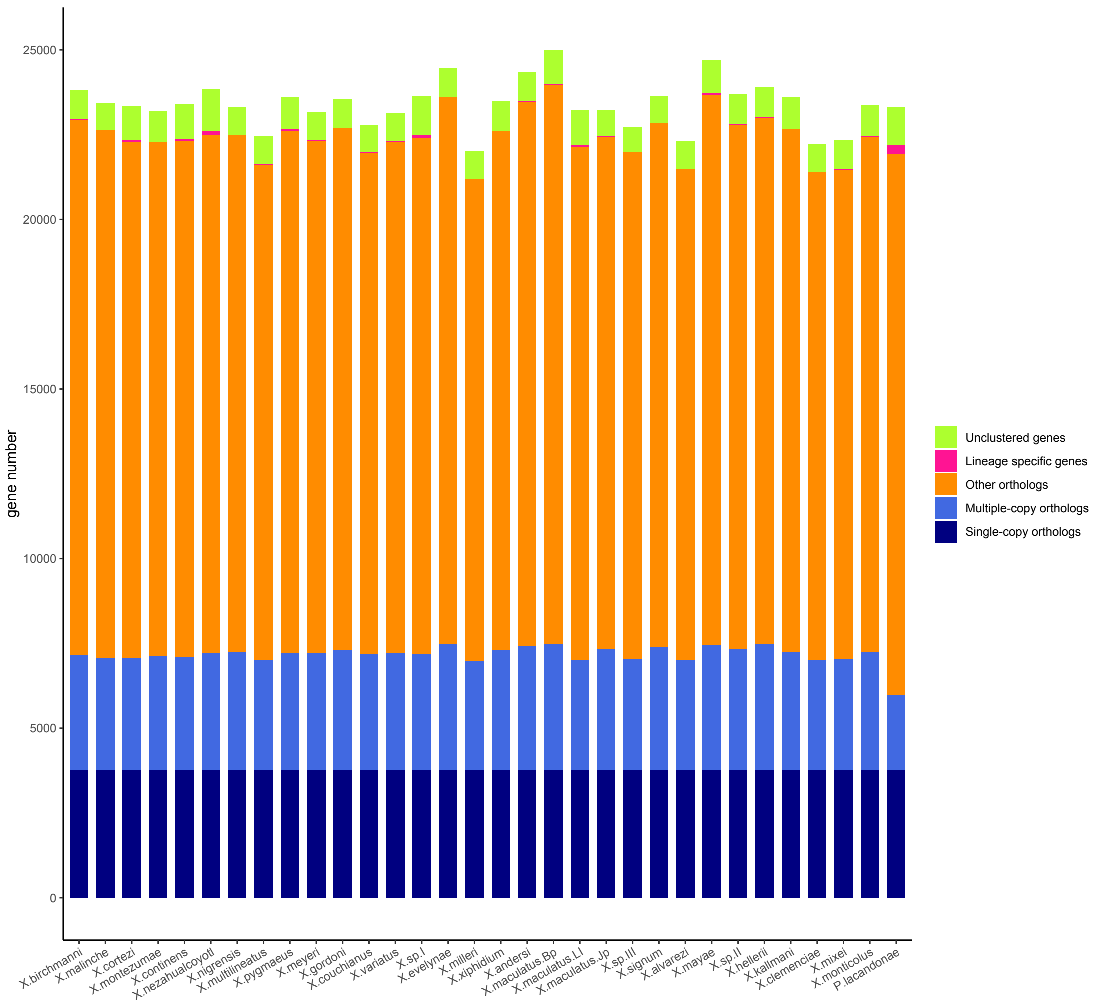

Supplementary figure S3. Bar plots showing the content of orthologous protein coding genes across species.

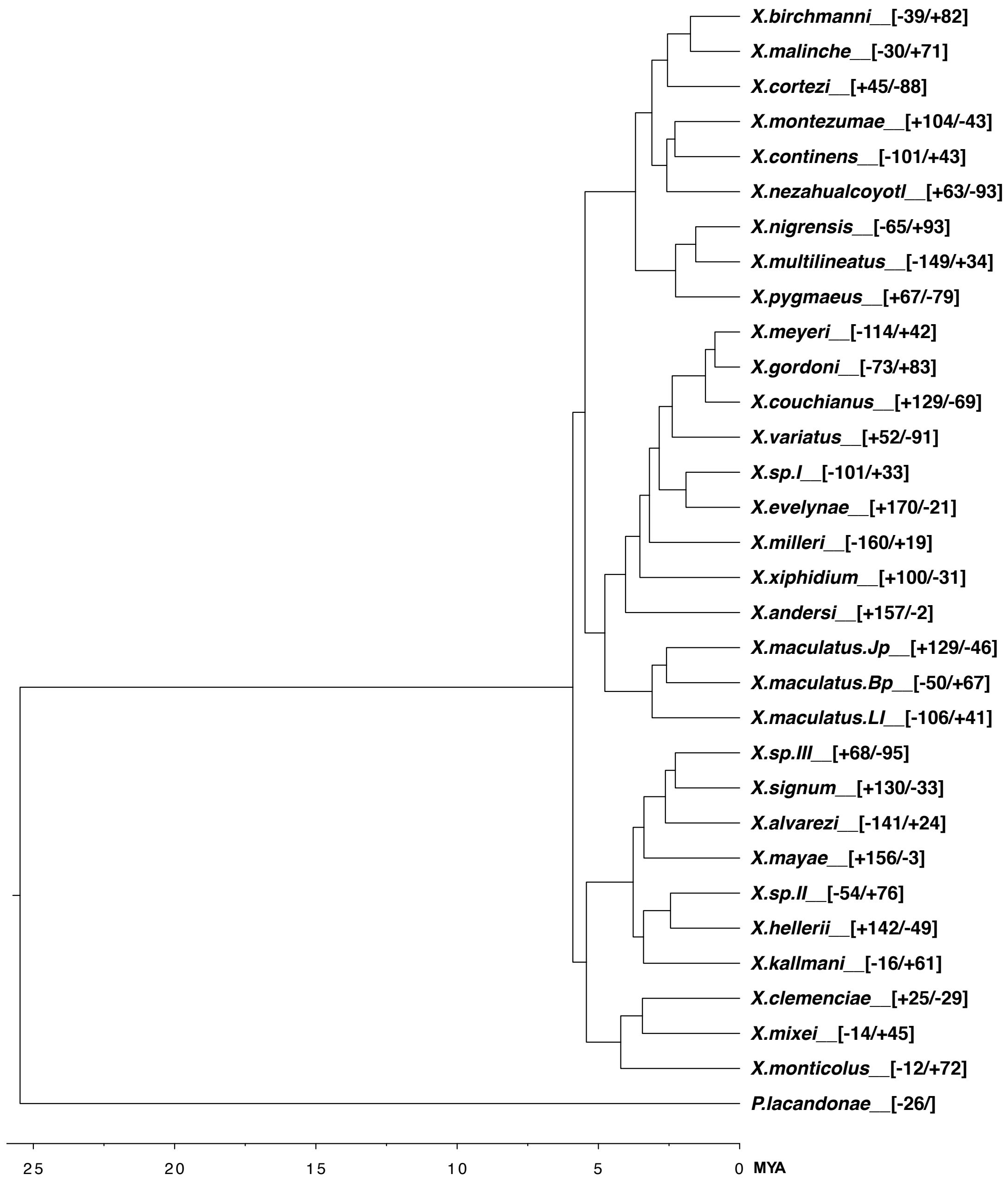

Supplementary figure S4. Phylogenetic tree displaying the time of branch divergence and gene family expansion and contraction in each lineage. Number after + is the number of significantly expanded gene family; after -, the significantly contracted.

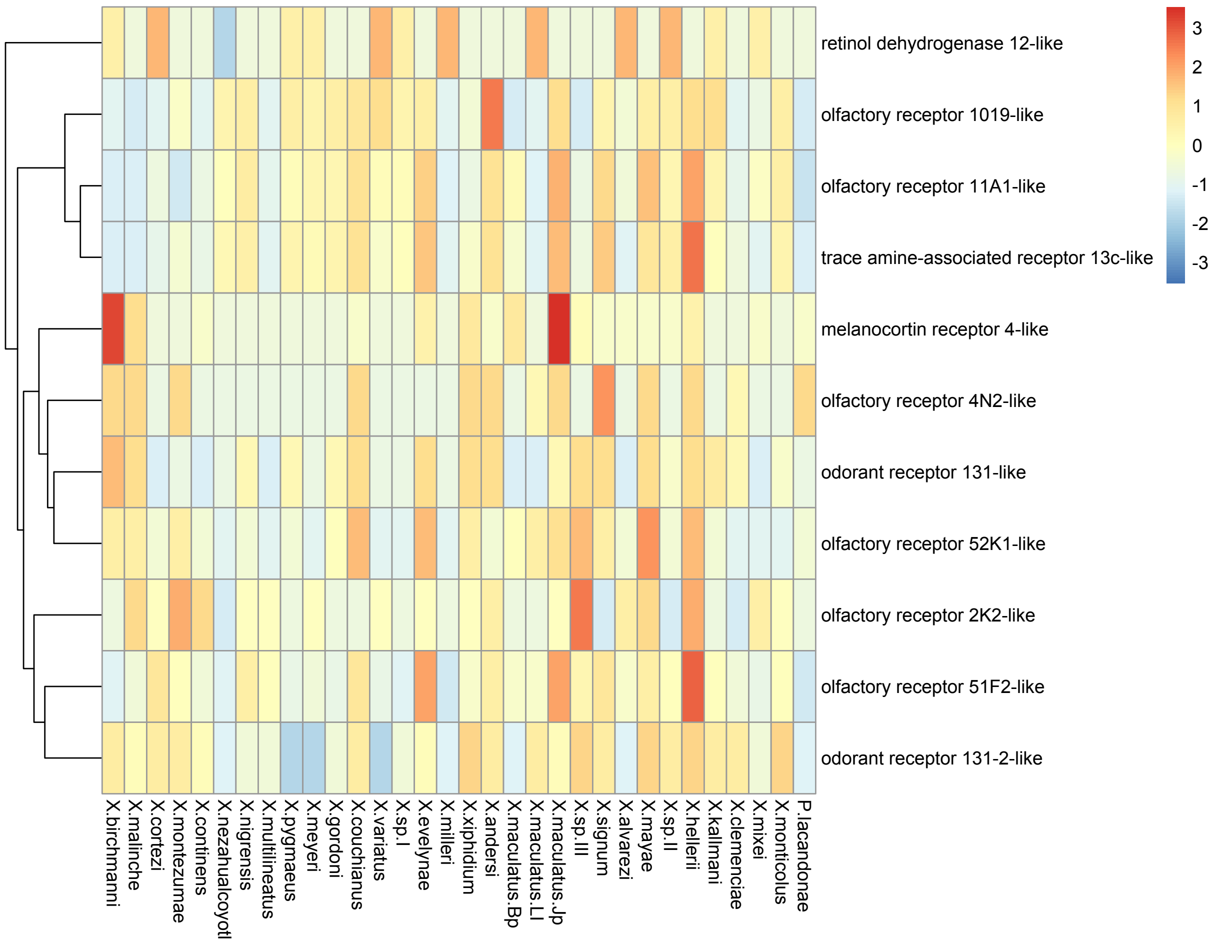

Supplementary figure S5. Heatmap displaying significant gene-familiy-size difference of olfactory receptors, odorant receptors, retinol dehydrogenase 12 and melanocortin 4 receptors (*mc4r*).

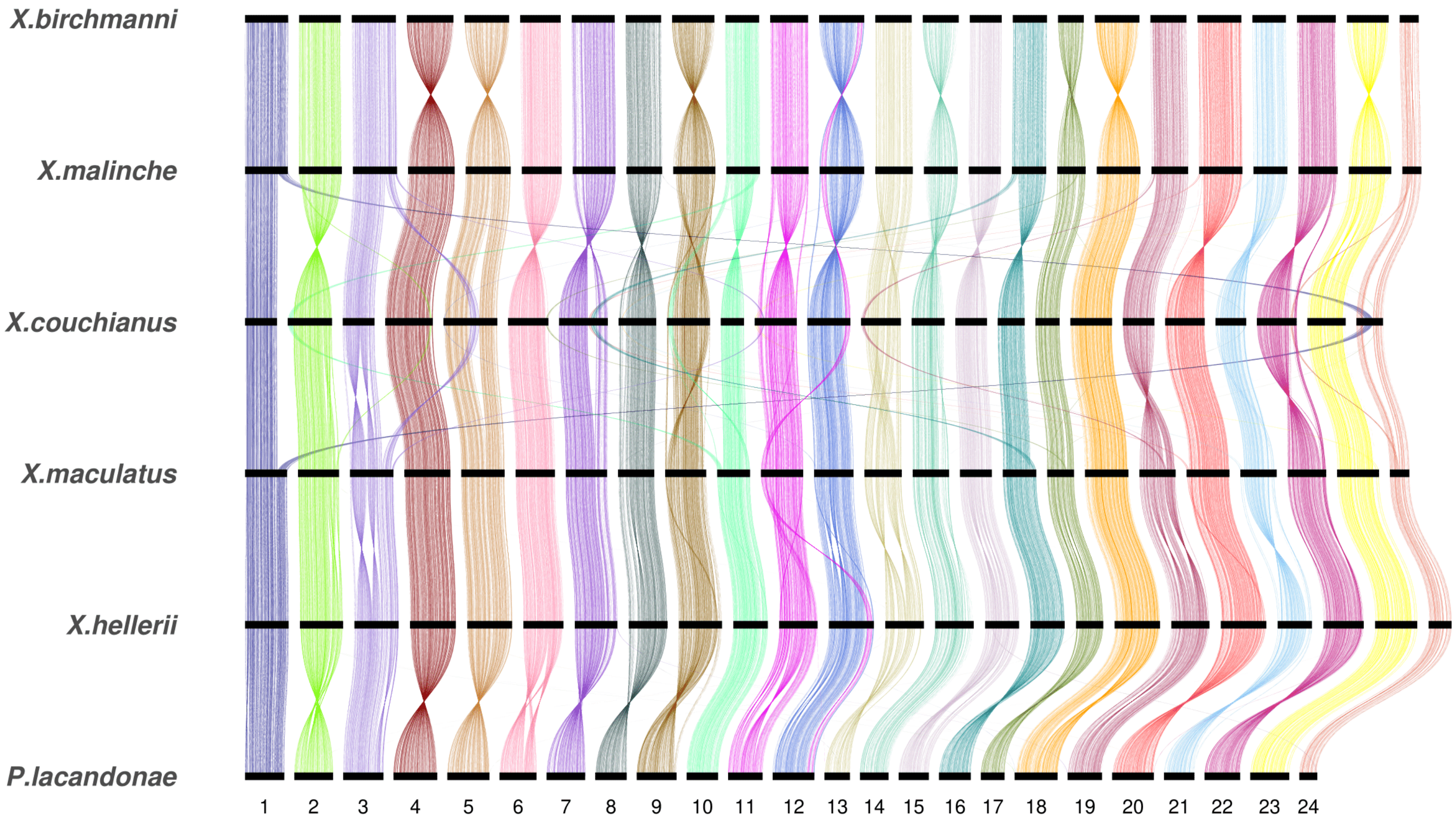

Supplementary figure S6. Sankey plots showing conserved synteny across the six species with chromosome-level assemblies. Black bars represent the chromosome, threads link the one-to-one orthologous genes across species

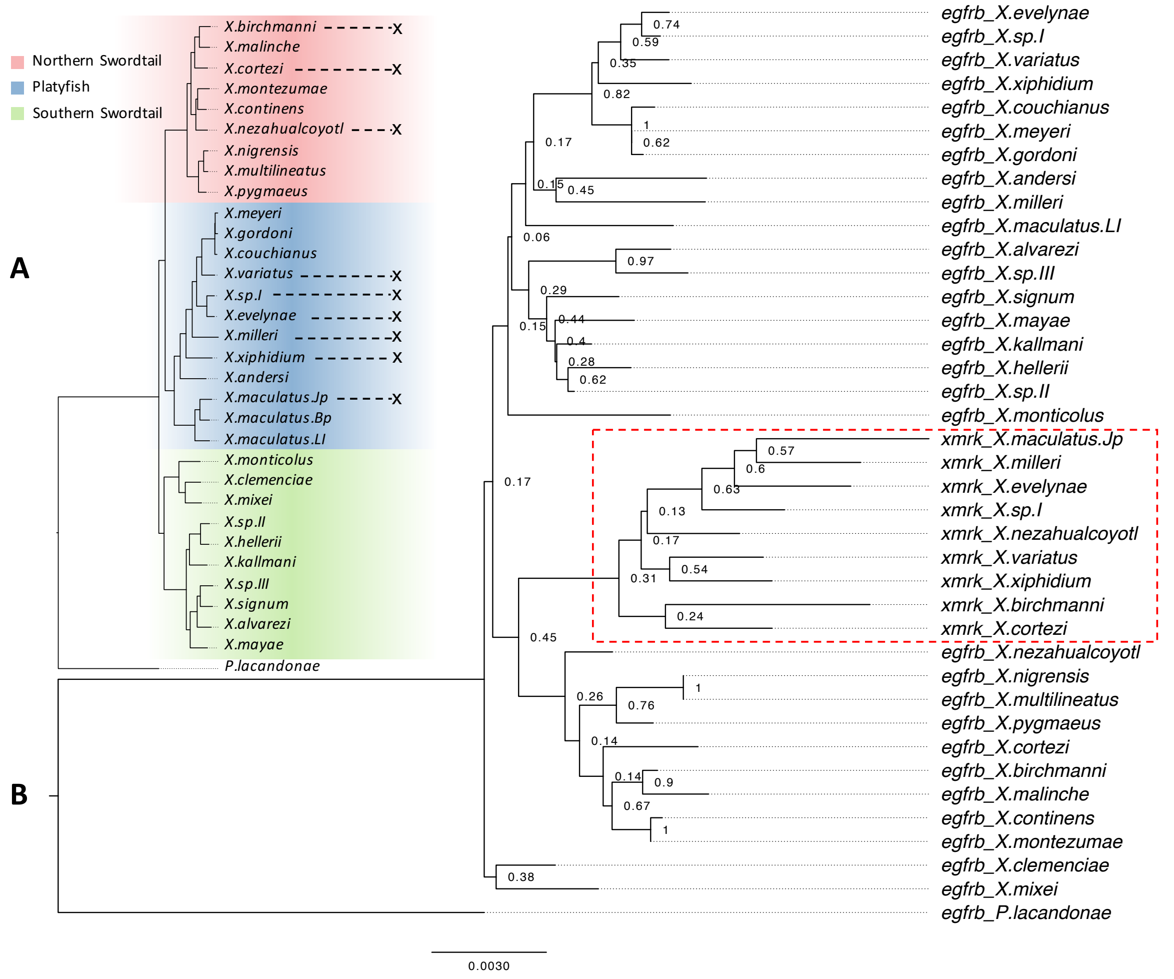

Supplementary figure S7. Identification of *xmrk* in *Xiphophorus*. (A) Retrieval of *xmrk* from *Xiphophorus* genomes (marked by X). **(B)** A Neighbor-joining (NJ) p-distance gene tree of *xmrk* constructed using coding sequence. Numbers are support value from 500 bootstraps.

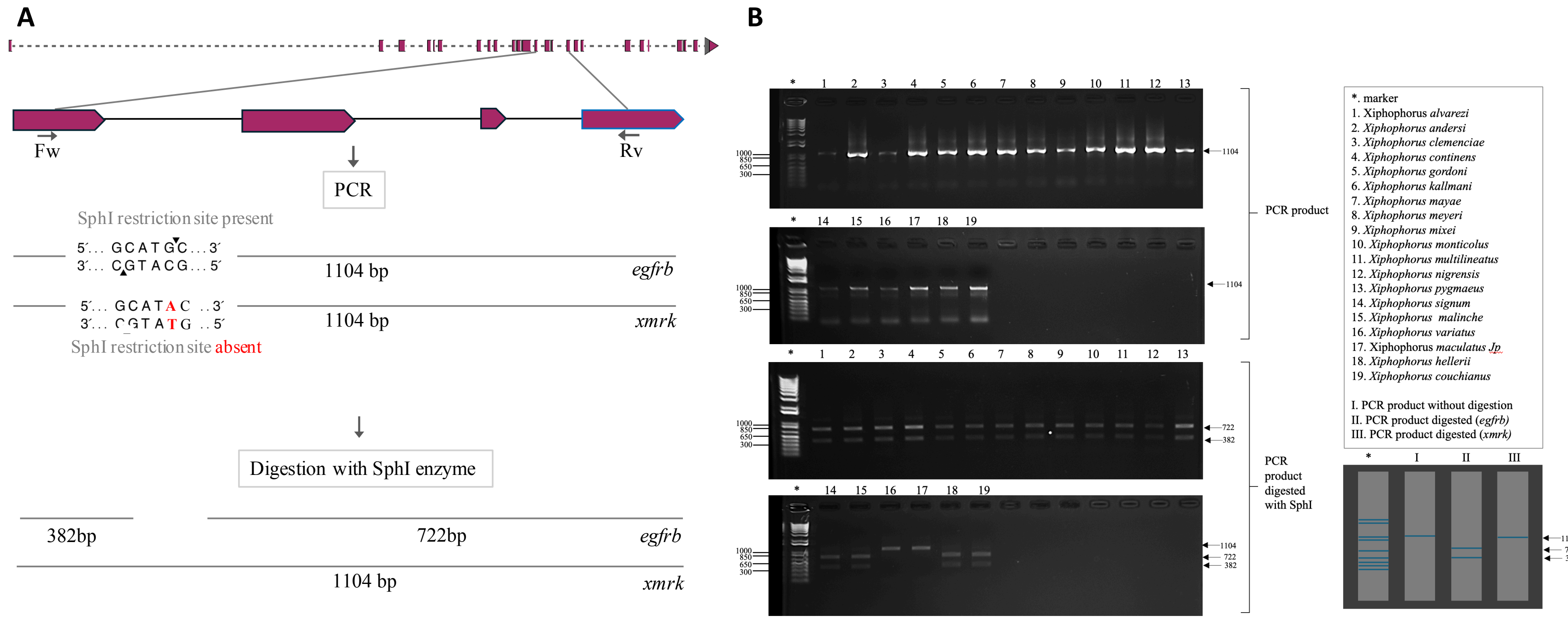

Supplementary figure S8. Confirmation of presence/absence of *xmrk* and *egrfb* genes using polymerase chain reaction (PCR) amplicon restriction emzyme digest (A) Scheme of the strategy used to discriminate *xmrk* from *egfrb* genes. Unlike *egfrb*, the PCR products of *xmrk* cannot be cut by SphI due to the absence of the restriction site. **(B)** Experiment results showing the presence (16: X. variatus and 17: X. maculatus Jp) and absence of *xmrk* (the rest).

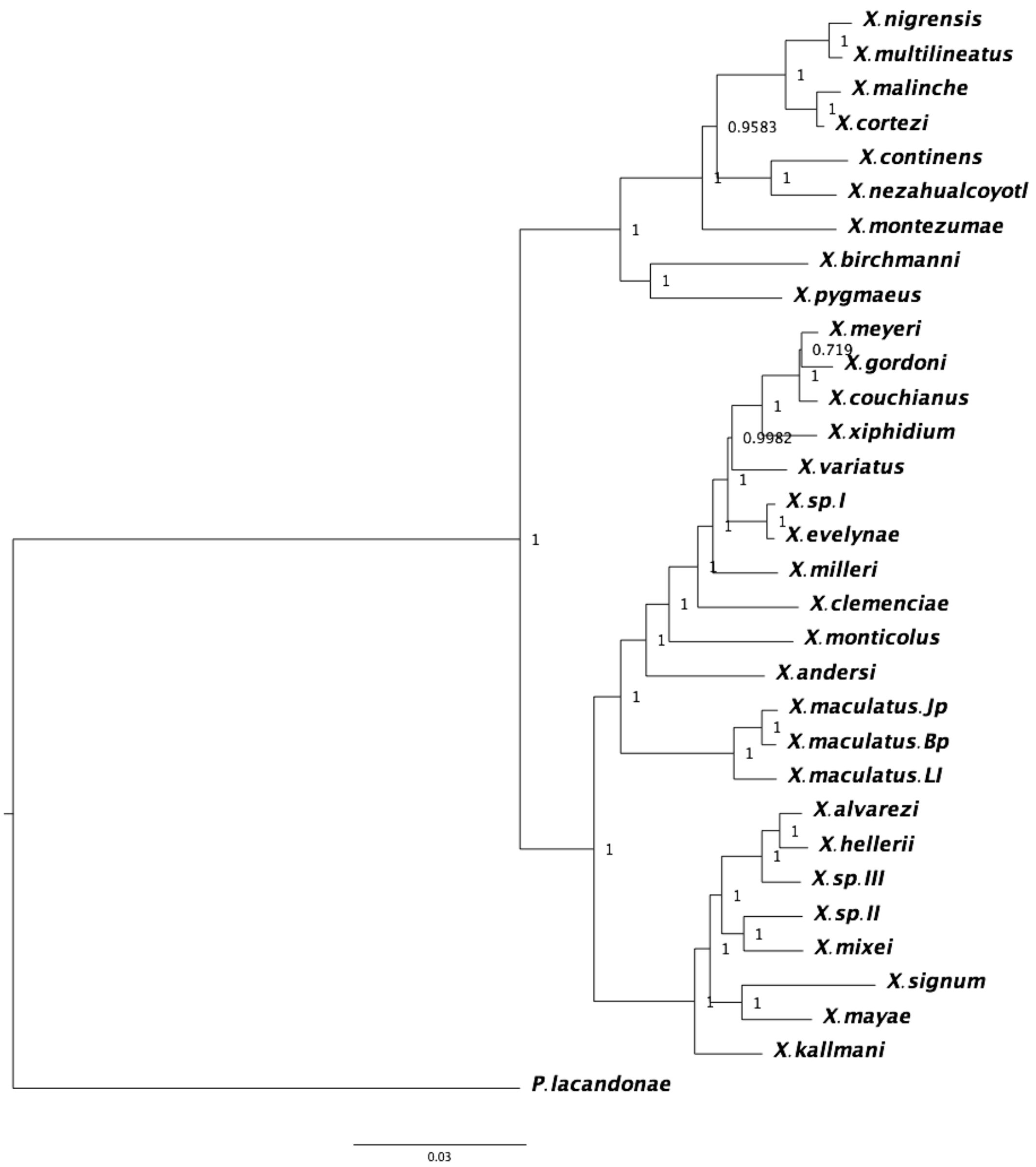

Supplementary figure S9. Mitochondrial phylogenetic tree constructed by using mitochondrial sequences with the method of bayesian inference.

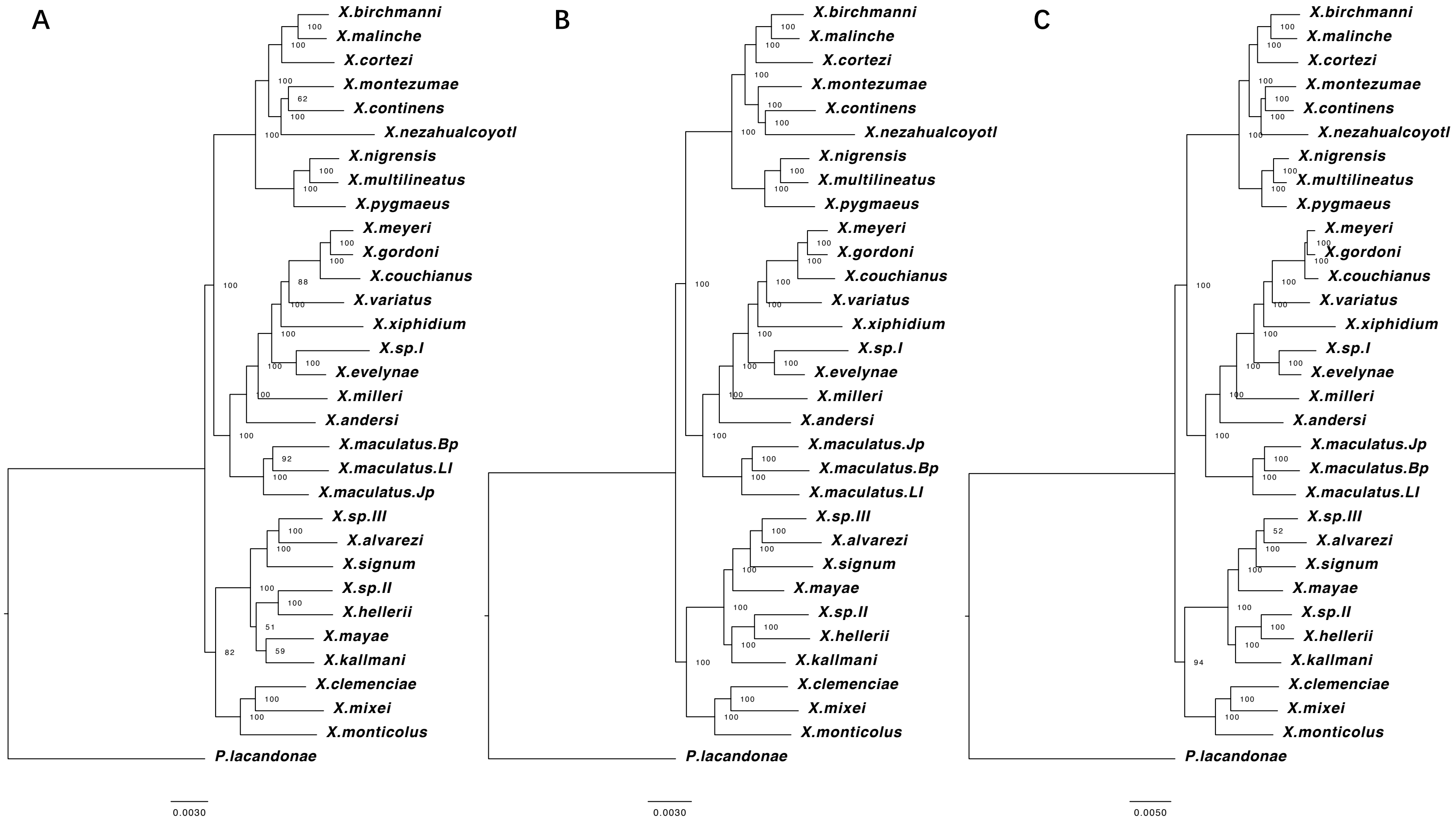

Supplementary figure S10. Phylogenetic trees using maximum-likelihood method based on protein sequences (A), coding sequences (B), and 4DTV sites (C) of 3,259 one to one orthologous genes. Numbers on the nodes represents bootstrap support values.

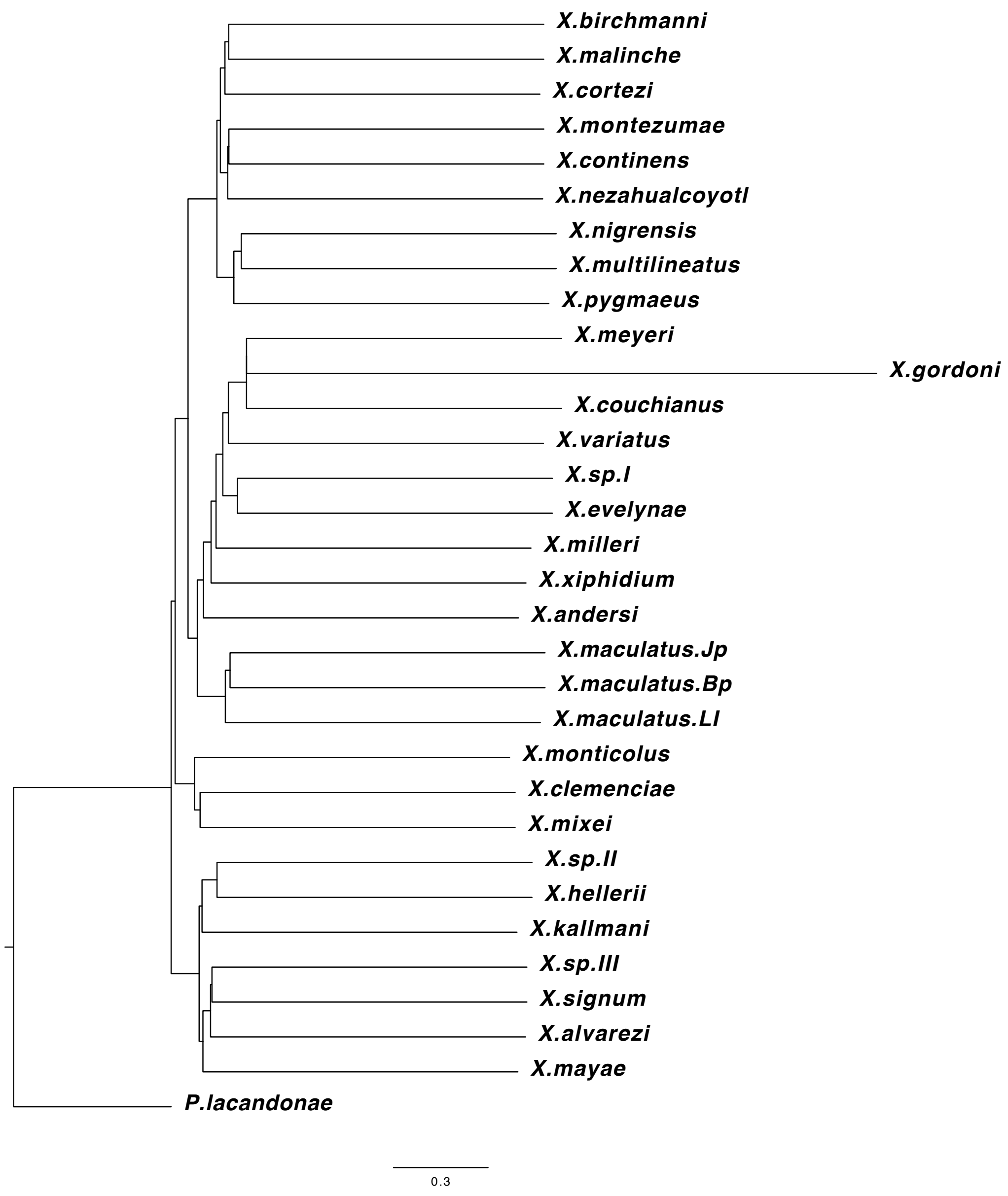

Supplementary figure S11. A coalescent phylogeny constructed based on whole genome alignments (~342Mb) using Coalescence-aware Alignment-based Species Tree EstimatoR (CASTER). CASTER is a site-based coalescent method that eliminates the need to predefine recombination-free loci.

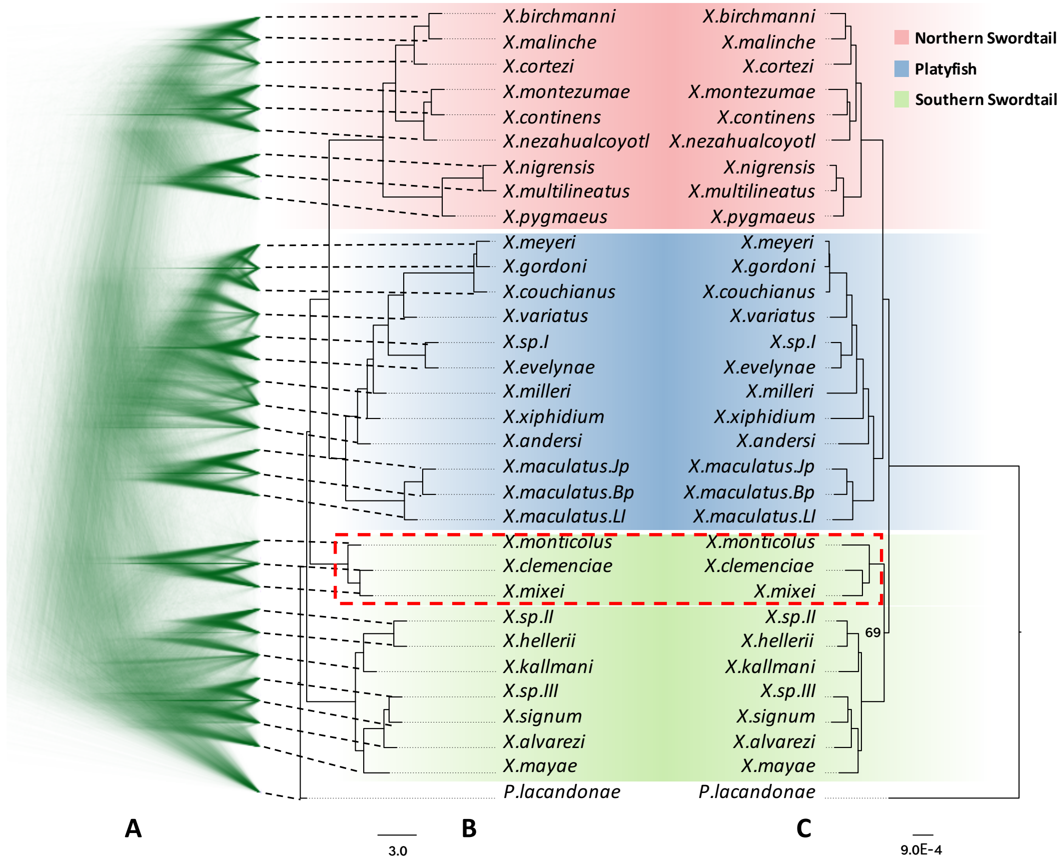

Supplementary figure S12. Phylogenetic trees based on CNEs showing incongruent topology resulting from coalescent- and concatenation-based methods. (A) Alignment of 6,766 trees made from 100 kb slide-windows showing phylogenetic discordance across the CNEs. **(B)** A coalescent phylogeny constructed using weightedASTRAL. **(C)** A concatenation-based phylogenetic tree constructed with maximum-likelihood method. Red frame indicates incongruent placement of the *X. monticolus*/*X. clemenciae*/*X. mixei* clade between coalescent- and concatenated-based phylogeny.

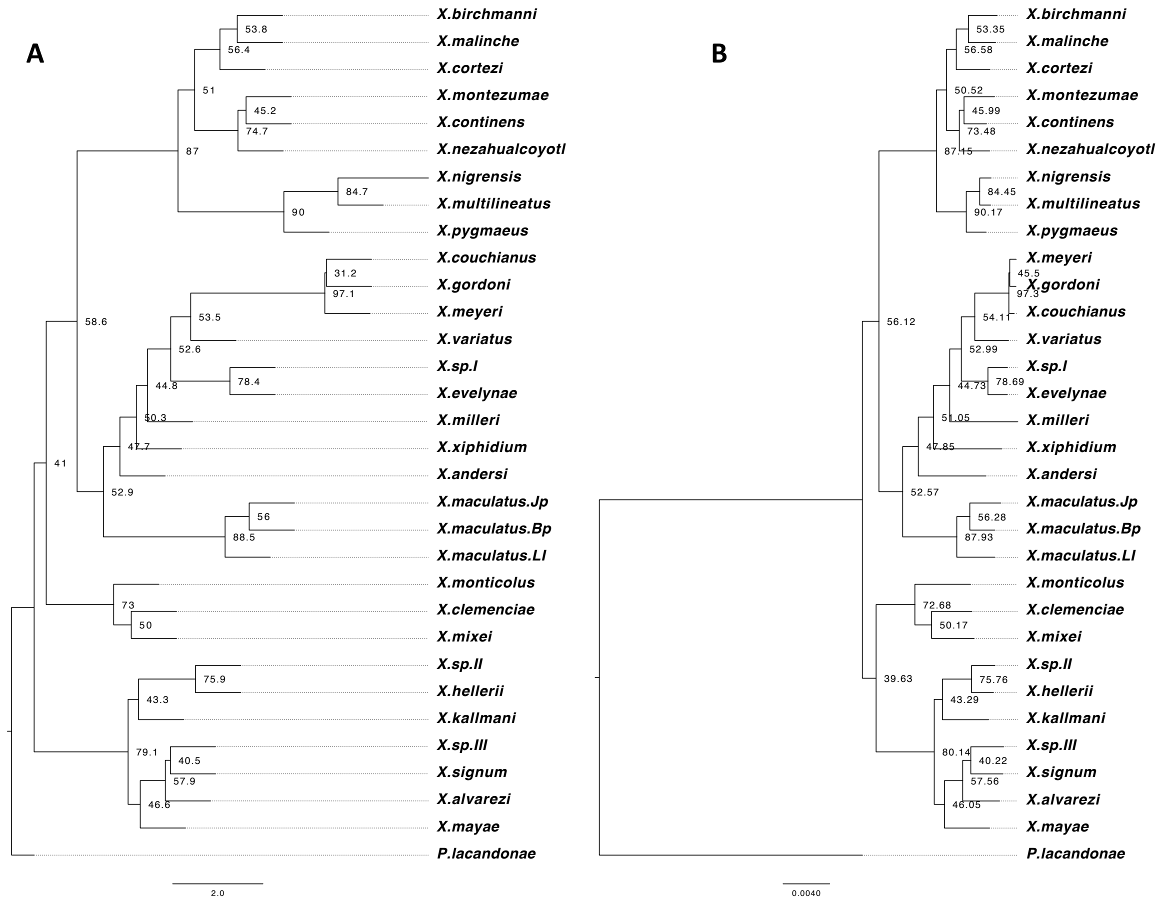

Supplementary figure S13. Coalescent-based phylogeny (A) and concatenated phylogeny with site concordant factor (sCF) shown on nodes. sCFs were calculated using IQ-TREE2 with whole genome alignment as input.

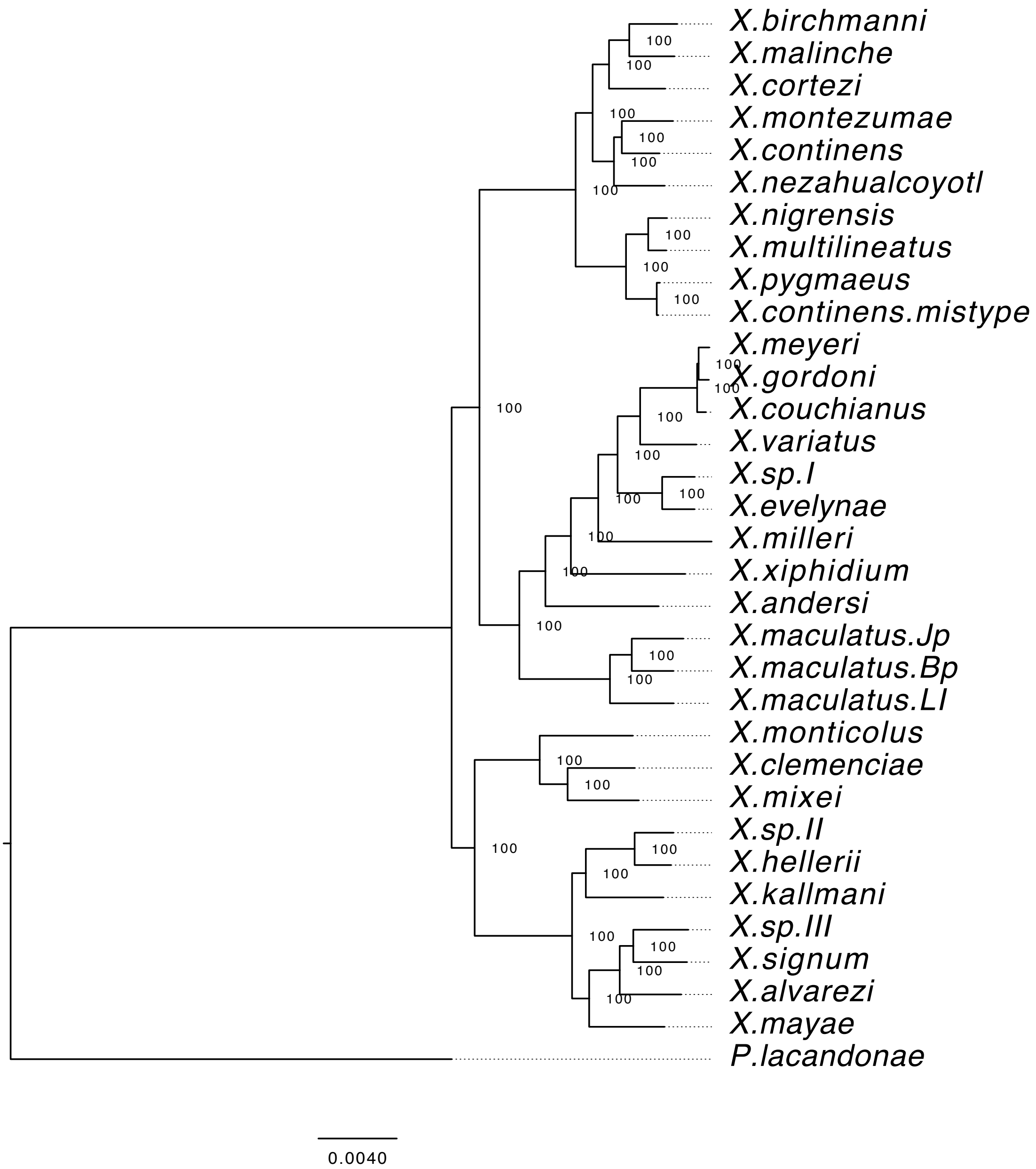

Supplementary figure S14. Phylogenetic tree revealing a *X. pygmaeus* was misidentified as *X. continens* in previous studies. The tree was reconstructed based on whole genome alignment using maximum-likelihood method. DNA of "*X.continens.mistype*" share the same resource from previous study Jones *et al*. 2013 and Cui *et al*. 2013. The WGS raw reads are available on SRA under accession number SRR29641096.

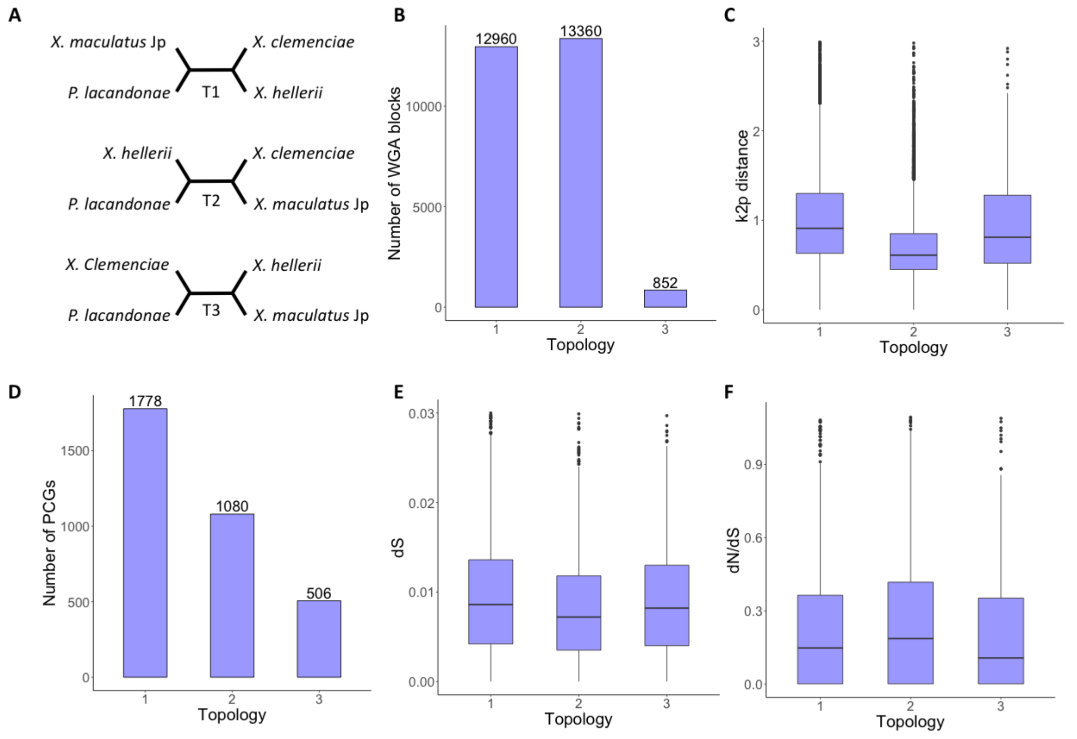

Supplementary figure S15. Incongruence phylogenies and evolutionary character of admixed genomic loci for hybridization between *X. clemenciae* and *X. maculatus*. (A) Phylogenetic topologies with T1 as the genome-wide consensus tree, T2 suggesting ILS or hybridization between *X. clemenciae* and *X. maculatus* and T3 suggesting ILS or hybridization between *X. hellerii* and *X. maculatus*. (B) Number of WGA regions past AU test yielding different topologies. (C) k2p distance between *X. clemenciae* and *X. monticolus* for WGA regions corresponding to different-topologies. (C) Number of PCGs past AU test yielding different topologie. (E) dS values of PCGs corresponding to different-topologies. (F) dN/dS values of PCGs corresponding to different-topologies.

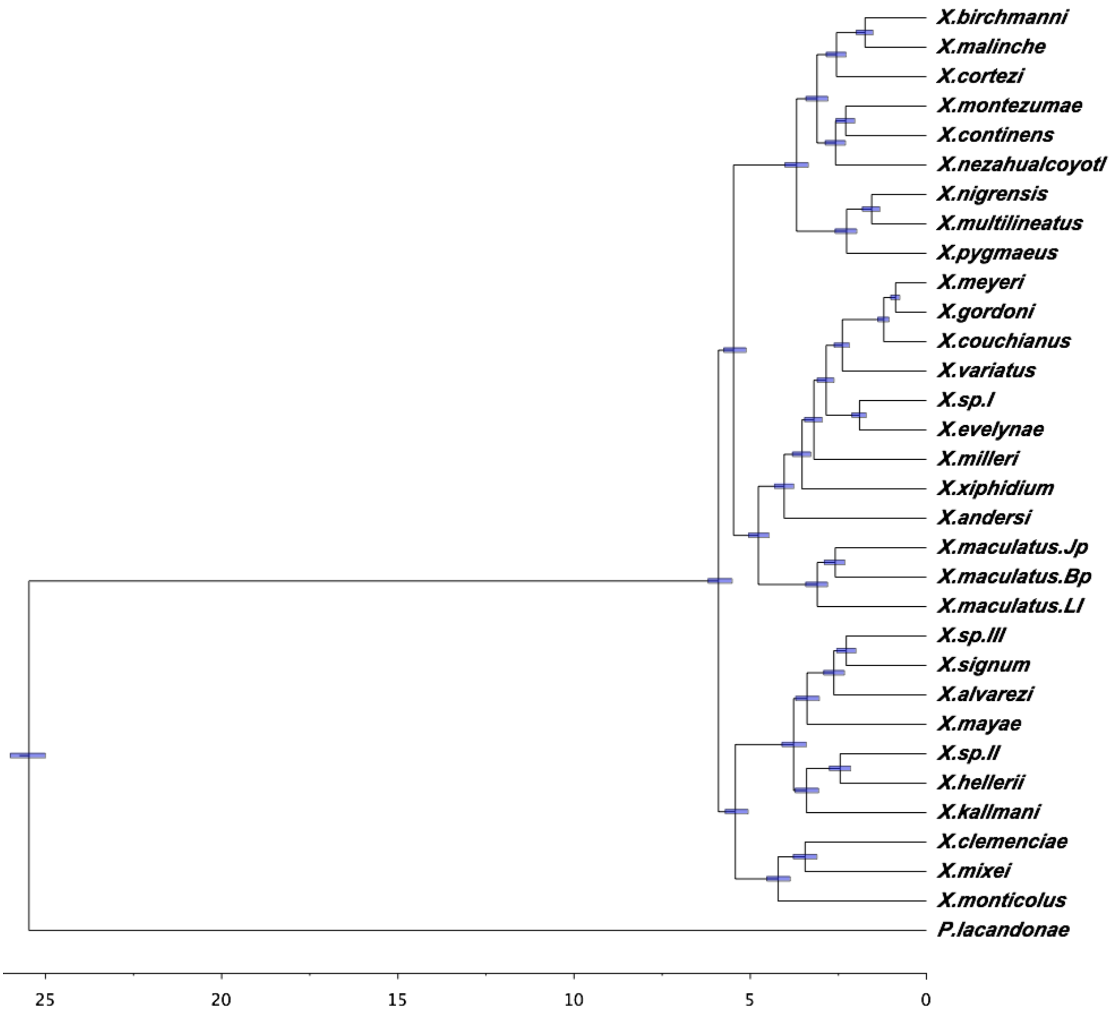

Supplementary figure S16. Phylogenetic tree showing the time estimates of branching events in the evolution of the genus *Xiphophorus*. Unit on x-axis is million years ago (MYA). Blue bar on each node represents the 95% confidence interval (CI) for the time estimation. Times were estimated using MCMCTree with time calibration for the root setting within 25-26 MYA and for the dividing of *Xiphophorus* within 4-6 MYA.
