# Supplementary material for: Phylogenomic analyses of all species of swordtail fishes (genus Xiphophorus) show that hybridization preceded speciation
Source: Nat Commun. 2024 Aug 4;15:6609. doi: 10.1038/s41467-024-50852-6 (PMC11298535; doi:10.1038/s41467-024-50852-6)
Supplement: Supplementary file 3 — Description of Additional Supplementary Files [file 41467_2024_50852_MOESM3_ESM.pdf]

Description of Additional Supplementary Files

File Name: Supplementary Data 1

Description: Tissue resource, technics and data of genome sequencing.

File Name: Supplementary Data 2

Description: Genome assembly metrics for each species.

File Name: Supplementary Data 3

Description: Density (%) of GC, small RNA and transposable element in the genome of each species.

File Name: Supplementary Data 4

Description: The coverage (%) of *Xiphophorus maculatus* assembly when it was mapped by assembly of each species.

File Name: Supplementary Data 5

Description: The sequence difference (%) between the genome of *Xiphophorus maculatus* and each other species.

File Name: Supplementary Data 6

Description: Annotation of protein coding genes for each species.

File Name: Supplementary Data 7

Description: The 356 gene families identified by CAFÉ analysis that underwent significant size change during the *Xiphophorus* evolution.
