# Supplementary material for: Phylogenomic analyses of all species of swordtail fishes (genus Xiphophorus) show that hybridization preceded speciation
Source: Nat Commun. 2024 Aug 4;15:6609. doi: 10.1038/s41467-024-50852-6 (PMC11298535; doi:10.1038/s41467-024-50852-6)
Supplement: Supplementary file 11 — Reporting Summary [file 41467_2024_50852_MOESM11_ESM.pdf]

nature portfolio

Corresponding author(s): Manfred Schartl

Last updated by author(s): 2024-6-27

Reporting Summary

Nature Portfolio wishes to improve the reproducibility of the work that we publish. This form provides structure for consistency and transparency in reporting. For further information on Nature Portfolio policies, see our Editorial Policies and the Editorial Policy Checklist.

Statistics

For all statistical analyses, confirm that the following items are present in the figure legend, table legend, main text, or Methods section.

n/a	Confirmed	
☐	☒	The exact sample size (*n*) for each experimental group/condition, given as a discrete number and unit of measurement
☐	☒	A statement on whether measurements were taken from distinct samples or whether the same sample was measured repeatedly
☐	☒	The statistical test(s) used AND whether they are one- or two-sided<br>*Only common tests should be described solely by name; describe more complex techniques in the Methods section.*
☐	☒	A description of all covariates tested
☐	☒	A description of any assumptions or corrections, such as tests of normality and adjustment for multiple comparisons
☐	☒	A full description of the statistical parameters including central tendency (e.g. means) or other basic estimates (e.g. regression coefficient) AND variation (e.g. standard deviation) or associated estimates of uncertainty (e.g. confidence intervals)
☐	☒	For null hypothesis testing, the test statistic (e.g. *F*, *t*, *r*) with confidence intervals, effect sizes, degrees of freedom and *P* value noted<br>*Give P values as exact values whenever suitable.*
☐	☒	For Bayesian analysis, information on the choice of priors and Markov chain Monte Carlo settings
☐	☒	For hierarchical and complex designs, identification of the appropriate level for tests and full reporting of outcomes
☐	☒	Estimates of effect sizes (e.g. Cohen's *d*, Pearson's *r*), indicating how they were calculated

Our web collection on statistics for biologists contains articles on many of the points above.

Software and code

Policy information about availability of computer code

Data collection	Genome data from NCBI were downloaded using GNU Wget 1.20.3, a non-interactive network retriever.
Data analysis	SOAPdenovo v2.04<br>SSPACE v3.0<br>Platanus v1.2.4<br>GapCloser v1.12<br>Supernova v2.1.1<br>Supernova v2.8.0<br>BUSCO v2.0.1<br>assemblathon_stats.pl<br>AGUSTUS v3.2.3<br>RepeatModeler<br>RepeatMasker<br>Exonerate v2.4.0<br>Genewise v2.2.0<br>GenblastA v1.0.4<br>fastp v0.23.4<br>HISAT v2.1.0<br>StringTie v2.2.0

Trinity v2.13.1
splign
BLAST+ v2.10.1
Hcluster_sg
TreeBeST v.0.5
CAFE v5
MITObim v1.9.1
norgal v1.0
MACSE v2
MUSCLE v5
Gblocks 0.91b
RAxML v8.2.10
MrBayes 3.2.7a
minimap2 v2.28
MULTIZ
DensiTree v2.0
ASTER
ASTRAL
IQ-TREE v2
phastCons
phyloFit
MCMCTree v4.2
Dsuite v0.5
SNaQ v2.2.2
Dendroscope v3
The in-house source code used in the manuscript can be obtained from https://github.com/dukecomeback/XiphoMicroEvo.

For manuscripts utilizing custom algorithms or software that are central to the research but not yet described in published literature, software must be made available to editors and reviewers. We strongly encourage code deposition in a community repository (e.g. GitHub). See the Nature Portfolio guidelines for submitting code & software for further information.

Data

Policy information about availability of data

All manuscripts must include a data availability statement. This statement should provide the following information, where applicable:

- Accession codes, unique identifiers, or web links for publicly available datasets
- A description of any restrictions on data availability
- For clinical datasets or third party data, please ensure that the statement adheres to our policy

Our data accession details are described in the manuscript under Data availability section.
Raw reads of the whole genome sequencing generated in the study have been deposited in SRA under accession number PRJNA972672. Assemblies and annotations are available in figshare under DOI https://doi.org/10.6084/m9.figshare.23596515.v1. Assemblies of X. maculatus, X. couchianus, X. helleri, X. birchmanni and X. malinche are from previously published sources. They are available in GeneBank under accession number GCA_002775205.2, GCA_001444195.3 and GCA_001443345.1 or figshare under DOI https://doi.org/10.6084/m9.figshare.23596515.v1

Research involving human participants, their data, or biological material

Policy information about studies with human participants or human data. See also policy information about sex, gender (identity/presentation), and sexual orientation and race, ethnicity and racism.

Reporting on sex and gender	Our research does not involve any human participants.
Reporting on race, ethnicity, or other socially relevant groupings	*Please specify the socially constructed or socially relevant categorization variable(s) used in your manuscript and explain why they were used. Please note that such variables should not be used as proxies for other socially constructed/relevant variables (for example, race or ethnicity should not be used as a proxy for socioeconomic status).*<br>*Provide clear definitions of the relevant terms used, how they were provided (by the participants/respondents, the researchers, or third parties), and the method(s) used to classify people into the different categories (e.g. self-report, census or administrative data, social media data, etc.)*<br>*Please provide details about how you controlled for confounding variables in your analyses.*
Population characteristics	*Describe the covariate-relevant population characteristics of the human research participants (e.g. age, genotypic information, past and current diagnosis and treatment categories). If you filled out the behavioural & social sciences study design questions and have nothing to add here, write "See above."*
Recruitment	*Describe how participants were recruited. Outline any potential self-selection bias or other biases that may be present and how these are likely to impact results.*
Ethics oversight	*Identify the organization(s) that approved the study protocol.*

Note that full information on the approval of the study protocol must also be provided in the manuscript.

Field-specific reporting

Please select the one below that is the best fit for your research. If you are not sure, read the appropriate sections before making your selection.

☒ Life sciences ☐ Behavioural & social sciences ☐ Ecological, evolutionary & environmental sciences

For a reference copy of the document with all sections, see nature.com/documents/nr-reporting-summary-flat.pdf

Life sciences study design

All studies must disclose on these points even when the disclosure is negative.

Sample size	Not applied cause in the study only one individual was sampled to represent each species for whole genome sequencing.
Data exclusions	No data excluded.
Replication	The main study was carried out using bioinformatic methods. Following the method with same version of softwares and parameters, the reproductivity will be achieved.
Randomization	Not relevant cause there is no experimental group allocated in the study.
Blinding	Not relevant cause there is no experimental group allocated in the study.

Reporting for specific materials, systems and methods

We require information from authors about some types of materials, experimental systems and methods used in many studies. Here, indicate whether each material, system or method listed is relevant to your study. If you are not sure if a list item applies to your research, read the appropriate section before selecting a response.

Materials & experimental systems

n/a	Involved in the study	
☒	☐	Antibodies
☒	☐	Eukaryotic cell lines
☒	☐	Palaeontology and archaeology
☐	☒	Animals and other organisms
☒	☐	Clinical data
☒	☐	Dual use research of concern
☒	☐	Plants

Methods

n/a	Involved in the study	
☒	☐	ChIP-seq
☒	☐	Flow cytometry
☒	☐	MRI-based neuroimaging

Animals and other research organisms

Policy information about studies involving animals; ARRIVE guidelines recommended for reporting animal research, and Sex and Gender in Research

Laboratory animals

The strain information of Laboratory animals are described in Supplementary table S1. and below:

X.cortezi Rio Axtla, lab strain WLC 1303 adult male
X.montezumae Rio Ojo Frio, lab strain XGSC Rascon adult female
X.continens Ojo frio, lab strain WLC#DNA511 adult male
X.nezahualcoyotl Rio Tamesi drainage, XGSC ID10104 adult, sex not determined
X.nigrensis Rio Choy, lab strain WLC 2915 adult male
X.multilineatus Rio Coy, lab strain WLC 2913 adult male
X.pygmaeus Rio Axtla, lab strain WLC 3015 adult male
X.meyeri Múzquiz, lab strain WLC3094 adult male
X.gordoni Laguna St. Tecla, lab strain WLC 118/XGSC adult male
X.variatus Ciudad Mante, lab strain WLC III/2 adult male
X.sp.I Rio Santa Ana, lab strain WLC6772 adult female
X.evelynae Necaxa, lab strain WLC1239 adult male
X.milleri Catemaco, lab strain WLC 1324 adult male
X.xiphidium Rio Purification, lab strain WLC 1293 adult male
X.andersi Rio Atoyac, lab strain WLC1258 adult male
X.maculatus.Bp Kate's Lagoon, Belize, lab strain XGSC BpII adult female
X.maculatus.LI Lago Izabal, lab strain WLC 4381 adult female

X.sp.III Rio Dolores, lab strain WLC 2530 adult female
X.signum Rio Chajmaic, lab strain WLC1280 adult female
X.alvarezi Ixcan, WLC#DNA 5779 adult male
X.mayae Rio Dulce, lab strain WLC1570 adult female
X.sp.II Arroyo de Carizal, lab strain WLC 5534 adult female
X.kallmani Catemaco, lab strain WLC 1354 adult male
X.clemenciae Puente Chino Luiz, lab strain WLC5328 adult female
X.mixei Rio del Sol, XGSC 7830 adult female
X.monticolus El Tejon, lab strain XGSC Tej adult female
P.lacandonae Misol-Ha, lab strain WLC 5281 adult female

Wild animals	No wild animals were included in the study
Reporting on sex	Sex was not considered in the study, however we included the information in Supplementary table S1.
Field-collected samples	No field-collected samples involved in this study.
Ethics oversight	Relevant protocol was approved by Institutional Animal Care and Use Committee (IACUC).

Note that full information on the approval of the study protocol must also be provided in the manuscript.

Plants

Seed stocks	*Report on the source of all seed stocks or other plant material used. If applicable, state the seed stock centre and catalogue number. If plant specimens were collected from the field, describe the collection location, date and sampling procedures.*
Novel plant genotypes	*Describe the methods by which all novel plant genotypes were produced. This includes those generated by transgenic approaches, gene editing, chemical/radiation-based mutagenesis and hybridization. For transgenic lines, describe the transformation method, the number of independent lines analyzed and the generation upon which experiments were performed. For gene-edited lines, describe the editor used, the endogenous sequence targeted for editing, the targeting guide RNA sequence (if applicable) and how the editor was applied.*
Authentication	*Describe any authentication procedures for each seed stock used or novel genotype generated. Describe any experiments used to assess the effect of a mutation and, where applicable, how potential secondary effects (e.g. second site T-DNA insertions, mosiacism, off-target gene editing) were examined.*
